# “Diagnostic Performance of Artificial Intelligence in Evaluating Tricuspid Regurgitation: A Systematic Review and Meta‐Analysis”

**DOI:** 10.1002/clc.70366

**Published:** 2026-06-05

**Authors:** Pooya Eini, Homa Serpoush, Mohammad Rezayee, Milan Kassulke

**Affiliations:** ^1^ Cardiovascular Imaging Research Center Rajaie Cardiovascular Institute Tehran Iran; ^2^ Hamadan University of Medical Sciences Hamadan Iran; ^3^ College of Human Medicine Michigan State University East Lansing Michigan USA

**Keywords:** artificial intelligence, echocardiography, electrocardiography, machine learning, tricuspid regurgitation

## Abstract

**Background:**

Tricuspid regurgitation (TR) is a common valvular heart disease affecting 0.55%–1.6% of adults, rising to 5%–8% in those over 75 years, often secondary to left‐sided pathology or pulmonary hypertension. Moderate‐to‐severe TR independently predicts mortality and heart failure hospitalization, yet underdiagnosis persists due to echocardiography's operator dependence and variability. Artificial intelligence (AI), including machine learning (ML) and deep learning (DL), promises automated detection to enhance sensitivity and reproducibility. This systematic review and meta‐analysis synthesizes evidence on AI's diagnostic performance for TR using echocardiographic or alternative modalities.

**Methods:**

Following PRISMA guidelines, we searched PubMed, Embase, Scopus, Web of Science, and EBSCO from inception to August 2025, without language restrictions. Eligibility used PICOS: adults with TR evaluation; AI/ML index tests; clinician‐interpreted echocardiography reference; outcomes, including AUROC, sensitivity, and specificity. Data extraction and quality assessment by two reviewers; random‐effects meta‐analysis for pooled estimates; heterogeneity via I^2^; certainty per GRADE.

**Results:**

Eight studies were included. Pooled AUROC was 0.89 (95% CI 0.86–0.92) for TR detection. Echocardiography‐based models showed sensitivity 0.87 (95% CI 0.81–0.90), specificity 0.88 (95% CI 0.73–0.95), AUROC 0.92 (95% CI 0.89–0.94); ECG‐based models had AUROC 0.805. Substantial heterogeneity (I^2^ > 90%) arose from modalities and reference standards; GRADE certainty moderate due to retrospective designs and limited external validation.

**Conclusions:**

AI demonstrates promising diagnostic accuracy for TR, potentially standardizing early detection and triage. However, heterogeneity and methodological gaps necessitate larger prospective, multicenter studies with standardized reporting (e.g., TRIPOD‐AI) to confirm clinical utility.

## Graphical abstract

Artificial intelligence demonstrates strong diagnostic accuracy for detecting tricuspid regurgitation, particularly with echocardiography‐based models. Despite promising performance, substantial heterogeneity and limited external validation highlight the need for prospective, multicenter studies before routine clinical implementation.

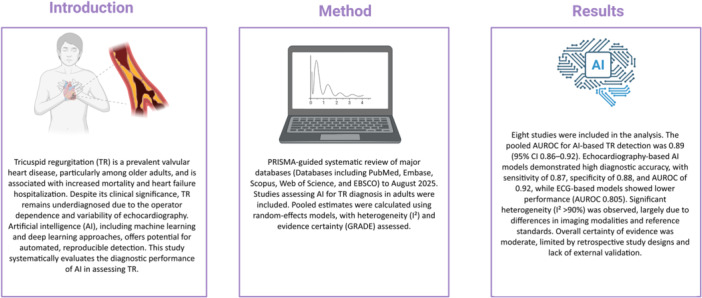

## 1 Introduction

Tricuspid regurgitation (TR) is a common yet frequently underdiagnosed valvular heart disease, most often arising secondarily from left‐sided cardiac pathology, pulmonary hypertension, or atrial fibrillation [1]. National screening and community studies demonstrate substantial geographic variability in tricuspid regurgitation prevalence ranging from 0.55% in the United States to 2.7% in older UK populations and indicate a sharp rise in TR‐related mortality since 2013 [2, 3]. The condition predominates in women and frequently coexists with heart failure or pulmonary hypertension [4]. Previous studies have shown that permanent pacemaker implantation, particularly with right ventricular apical lead positioning, is associated with the development or progression of tricuspid regurgitation due to mechanical leaflet interference, altered right ventricular geometry, and pacing‐induced dyssynchrony [5].

Echocardiography (transthoracic or transesophageal) remains the primary diagnostic modality, yet detection of TR particularly mild or subtle regurgitant jets, is hampered by variable image quality, weak Doppler signals from right‐sided flows, operator dependence, and interobserver disagreement. These factors contribute to under‐recognition in routine clinical practice. Many cases escape detection or timely management due to a gradual onset and nonspecific symptoms. As TR progresses, it drives right ventricular dilation, atrial enlargement, hepatic congestion, and reduced survival, underscoring the critical need for reliable early detection [6].

Artificial intelligence (AI), including machine learning (ML) and deep learning (DL), offers promise in cardiovascular disorders detection and risk prediction [7]. AI has already proven effective in echocardiography for view classification, chamber segmentation, and flow analysis, providing a foundation for valvular assessment [8]. However, AI applications for TR detection remain limited compared with left‐sided valves, largely due to anatomical complexity and lower signal‐to‐noise ratios in tricuspid imaging. Emerging AI tools could enhance screening efficiency, support population‐level surveillance, and facilitate timely referral for confirmatory grading or intervention [9].

No prior meta‐analysis has synthesized AI models specifically for TR detection. This systematic review and meta‐analysis synthesizes current evidence on the diagnostic performance of AI and ML approaches for detecting TR using echocardiographic and ECG modalities. By assessing detection accuracy, robustness, and reproducibility, the study addresses key barriers in conventional TR recognition and evaluates the potential of AI to standardize and scale early detection in clinical workflows.

## 2 Methods

### 2.1 Study Design and Registration

This systematic review and meta‐analysis followed the Preferred Reporting Items for Systematic Reviews and Meta‐Analyses for Diagnostic Test Accuracy (PRISMA) guidelines. The protocol was prospectively registered in PROSPERO (registration number: CRD420251253366) [10]. No amendments to the protocol were implemented post‐registration to maintain adherence to the predefined methodology.

### 2.2 Search Strategy

A comprehensive literature search was conducted across PubMed, Embase, Scopus, Web of Science, and Cochrane Library from database inception to August 2025. The search strategy combined controlled vocabulary terms (e.g., MeSH and Emtree) with free‐text keywords for the disease (“tricuspid regurgitation,” “TR,” “tricuspid insufficiency,” and “right‐sided valvular disease”) and methods (“artificial intelligence,” “machine learning,” “deep learning,” “neural networks,” “AI,” and “automated quantification”). Boolean operators (AND/OR) were applied to combine concepts, with no restrictions on language or publication year. The full reproducible search syntax is provided in Supporting Table 1.

### 2.3 Eligibility Criteria (PICOS Framework)

Eligibility was defined using the Population, Index Test, Comparator/Reference Standard, Outcomes, and Study Design (PICOS) framework. The population included adult patients (aged ≥ 18 years) who underwent imaging or signal‐based evaluation for primary or secondary TR. The index test comprised AI‐based algorithms (ML or DL) for automated or semi‐automated TR severity grading. The reference standard consisted of clinician‐interpreted or guideline‐based TR grades derived from 2D/3D echocardiography, Doppler, or cardiac magnetic resonance imaging.

The reference standard was clinician‐interpreted echocardiography performed according to contemporary guideline recommendations (American Society of Echocardiography/European Association of Cardiovascular Imaging). We extracted information on whether reference standard assessors were blinded to AI model outputs; studies were rated as having lower risk of bias when blinding was explicitly reported. TR severity grading was accepted as reported by study authors, with harmonization to moderate‐or‐greater severity as described above.

For ECG‐based studies, we distinguished between diagnostic models (detecting concurrent TR at the time of ECG acquisition).

Outcomes encompassed diagnostic performance metrics, including area under the receiver operating characteristic curve (AUROC), sensitivity, specificity, precision, F1‐score, or data allowing construction of 2 × 2 contingency tables. Study designs were limited to original research with validation (internal or external); purely developmental studies without performance evaluation were excluded. Further exclusions applied to animal or in vitro studies, case reports, reviews, editorials, studies with insufficient data, non‐standard TR definitions, or duplicates.

### 2.4 Data Extraction

Two independent reviewers extracted data using a standardized Excel template. Extracted items included study characteristics (author, year, design, setting, and country), model details (AI/ML/DL type, imaging modality, and input features), population characteristics (sample size, demographics, TR etiology, and comorbidities), and performance metrics (AUROC, sensitivity, specificity, accuracy, and calibration). When multiple models were reported, the best‐performing or primary model was prioritized. Disagreements were resolved by consensus or third‐reviewer adjudication.

### 2.5 Risk of Bias and Certainty Assessment

Risk of bias was evaluated using the Prediction model Risk Of Bias Assessment Tool extended for AI (PROBAST + AI) across domains of participants, predictors, outcomes, and analysis [11]. The certainty of evidence was graded using the Grading of Recommendations Assessment, Development, and Evaluation for Diagnostic Test Accuracy (GRADE‐DTA) framework, with downgrading for bias, heterogeneity, indirectness, or imprecision. Assessments were performed independently by two reviewers, with conflicts resolved through discussion.

### 2.6 Statistical Analysis

Analyses were performed in R (version 4.5) using the mada and metafor packages. A bivariate random‐effects model was used to estimate the pooled sensitivity and specificity, along with 95% confidence intervals. For studies reporting multiple severity thresholds, we extracted data corresponding to this clinically relevant cutpoint. When studies used different grading schemes (e.g., 3‐grade vs. 4‐grade systems), we harmonized classifications to align with moderate‐to‐greater severity based on reported definitions. When studies reported multiple AI models or algorithmic variations, we used a predefined hierarchy to select one model per study for the primary analysis to avoid unit‐of‐analysis errors. The hierarchy prioritized: (1) externally validated models over internally validated models; (2) deep learning models over traditional machine learning models; (3) models using the most comprehensive input data (e.g., multiview echocardiography over single‐view); and (4) the model explicitly designated by authors as their primary or best‐performing model. Sensitivity analyses explored the impact of model selection by using the worst‐performing model from each study.

Pooled AUROC values were extracted directly from study reports when available. Overall AUROC was calculated from sensitivity and specificity using the DerSimonian–Laird random‐effects model in the metafor package.

The diagnostic odds ratio (DOR) was calculated as (sensitivity/(1‐sensitivity))/((1‐specificity)/specificity), and log DOR served as the effect size measure in meta‐regression analyses.

Heterogeneity was assessed with I^2^ and τ^2^ statistics; I^2^ > 50% indicated substantial heterogeneity. Given the anticipated heterogeneity in AI model architectures, study populations, and TR definitions, pooled estimates were considered exploratory and interpreted with caution. Subgroup analyses were conducted by imaging modality (echocardiography vs. ECG), algorithm type (DL vs. traditional ML), and validation method (internal vs. external). Meta‐regression explored sources of heterogeneity, including sample size, disease prevalence, and external validation status. Publication bias was examined using Deeks' funnel plot asymmetry test (*p* < 0.10 indicating potential bias). Sensitivity analyses (leave‐one‐out and restriction to peer‐reviewed articles excluding conference abstracts) evaluated robustness. Forest plots and hierarchical summary receiver operating characteristic (HSROC) curves were generated for visualization.

## 3 Results

### 3.1 Study Selection

The systematic search across five databases identified a total of 667 records: PubMed (87), Web of Science (115), Scopus (219), EMBASE (243), and the Cochrane Library (3). After removing 329 duplicates, 338 records were screened by title and abstract, resulting in 17 articles selected for full‐text review.

Of these, nine studies were excluded for specific reasons: one focused on mortality prediction, one study protocol, one evaluated atrial function in severe tricuspid regurgitation, one addressed diastolic function grading, one assessed tricuspid valve flow, one predicted post–septal myectomy outcomes, one examined mortality, one evaluated tricuspid valve morphology, and one assessed risk phenogroups among patients with TR.

**Figure 1:**
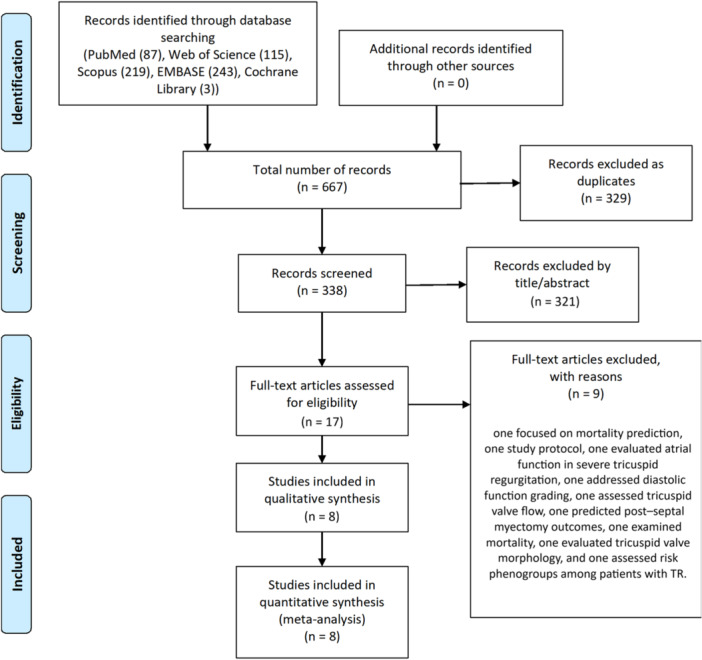
PRISMA flow diagram for study selection.

Finally, eight studies met the inclusion criteria. Among these, eight studies reported sufficient data and were included in the meta‐analysis (Figure 1).

### 3.2 Baseline Characteristics

Baseline characteristics are in Table 1. The eight included studies comprised more than 1.3 million echocardiographic studies and electrocardiograms from over 630 000 unique patients across the United States, China, Taiwan, Israel, France, and the United Kingdom, reflecting a broad spectrum of academic tertiary centers and community hospitals. Sample sizes ranged from 1541 transthoracic echocardiograms (Cohen 2025) to 988 618 ECGs (Liang 2025). Mean age was consistently in the seventh decade (60–76 years), with male representation of 48%–54%. Comorbidities were prevalent and typical of valvular heart disease populations: hypertension (40%–65%), diabetes mellitus (21%–36%), coronary artery disease (23%–41%), heart failure (12%–24%), atrial fibrillation (6%–13%), and chronic kidney disease (24%–44%). Event rates varied substantially across these datasets, with mild TR ranging from 5% to 36%, moderate TR from 6% to 22%, and severe TR from 1% to 28% and 14%–50% in echocardiography datasets intentionally enriched for valvular pathology.

**Table 1:** Baseline characteristics.

First author/year	Country	Validation	Modality	Selected features	Sample size	Mean age	Male%	Past medical history	Event rate	Machine learning algorithm
Long/2024 [12]	USA	Internal	Echocardiography (TTE)	Color Doppler video clips	93 065 TTEs (train 65 301; validation 14 018; test 13 746)	NR	NR	NR	Mild TR (24%), moderate TR (7%), severe TR (1%)	Hybrid neural network (CNN + transformer)
Xie/2024 [13]	China	Internal and external	Continuous wave (CW) Doppler spectra Echocardiography	CW Doppler spectra images	11 654 patients (development); 1500 internal + 573 external validation	NR	NR	NR	Mild TR (5%), moderate TR (10%), severe TR (8%)	DL
Cohen/2025 [14]	Israel, USA	External	Echocardiography (TTE)	Echocardiographic images	1541 TTEs	76	50.7	CAD (23.5%); Arrhythmia (78.5%); TIA (36.2%); Dementia (6.7%); PVD (42.3%); Chronic pulmonary disease (38.6%); Pulmonary circulation disorder (17.1%); RF (45%); Liver disease (3.4%); DM (21.5%); HTN (64.8%).	Mild TR (23%), moderate TR (22%), severe TR (28%)	DL
Long/2025 [15]	USA	Internal	Echocardiography (TTE)	Color Doppler videos	71 660 TTEs	61.4	48.5	NR	Mild TR (23%), moderate TR (12%), severe TR (2%)	DELINEATE‐Regurgitation (multiview CNN/transformer)
Vrudhula/2025 [16]	USA	Internal and external	Echocardiography	Apical 4‐chamber color Doppler videos	55 323 studies (development 47 312; internal validation 2462; external validation 5549)	Development 64.0 ± 17.4, Tuning 68.1 ± 16.3, Internal validation 63.4 ± 16.6, External validation 65.8 ± 18.1.	Development 53.8, Tuning 52.9, Internal validation 50.7, External validation 49.7.	DM 23.7%, 35.8%, 29.6%, 31.0%; HTN 40.3%, 58.2%, 51.9%, 55.1%; HLP 29.9%, 44.8%, 41.0%, 44.1%; CKD 24.1%, 43.8%, 24.0%, 24.5%; CAD 27.8%, 40.8%, 30.8%, 30.9%; HF 13.2%, 23.6%, 12.2%, 12.5%; Afib 6.7%, 12.7%, 6.4%, 6.4%; COPD 12.6%, 21.9%, 19.7%, 23.5% for the development, tuning, internal validation, and external validation sets, respectively.	Mild TR (36%), moderate TR (6%), severe TR (6%)	Deep learning computer vision (view classifier + severity classifier)
Lin/2024 [17]	Taiwan	Internal and external	ECG	ECG signals	77 047 patients	Development 64.0 ± 17.4, Tuning 68.1 ± 16.3, Internal validation 63.4 ± 16.6, External validation 65.8 ± 18.1.	Development 53.8%, Tuning 52.9%, Internal validation 50.7%, External validation 49.7%.	DM 23.7%, 35.8%, 29.6%, 31.0%; HTN 40.3%, 58.2%, 51.9%, 55.1%; HLP 29.9%, 44.8%, 41.0%, 44.1%; CKD 24.1%, 43.8%, 24.0%, 24.5%; CAD 27.8%, 40.8%, 30.8%, 30.9%; HF 13.2%, 23.6%, 12.2%, 12.5%; Afib 6.7%, 12.7%, 6.4%, 6.4%; COPD 12.6%, 21.9%, 19.7%, 23.5% for the development, tuning, internal validation, and external validation sets, respectively.	60%	DL
Cinq‐Mars/2025 [18]	France, USA	Internal	ECG	ECG signals	27 689 patients	65.9 ± 16.6	49%	NR	8%	CNN
Liang/2025 [19]	China, USA, UK	Internal and transnational external	ECG	ECG signals	988 618 ECGs (400 882 patients)	60.2	53	HTN (61.4%); Previous MI (14.2%); Smoker (17.9%); DM (28.6%); HLP (56.8%).	6%	CNN

Abbreviations: Afib, atrial fibrillation; CAD, coronary artery disease; CKD, chronic kidney disease; CNN, convolutional neural network; COPD, chronic obstructive pulmonary disease; CW, continuous‐wave Doppler; DELINEATE, deep learning echocardiography model using multiview CNN/transformer; DL, deep learning; DM, diabetes mellitus; ECG, electrocardiogram; HF, heart failure; HLP, hyperlipidemia; HTN, hypertension; Hybrid network, CNN–transformer combination model; MI, myocardial infarction; PVD, peripheral vascular disease; RF, renal failure; TIA, transient ischemic attack; TR, tricuspid regurgitation; Transformer, attention‐based deep learning architecture; TTE, transthoracic echocardiography.

Regarding machine learning architectures, all studies employed contemporary deep learning approaches. Five studies utilized echocardiography‐based convolutional neural networks (CNNs), often enhanced with advanced components: Long 2024 and Long 2025 (DELINEATE‐Regurgitation) implemented hybrid CNN–transformer models leveraging spatiotemporal features from multiview color Doppler videos; Vrudhula 2025 combined a view‐classification CNN with a severity‐classification CNN on apical‐4‐chamber color Doppler clips; Xie 2024 applied an end‐to‐end CNN to segmented continuous‐wave Doppler spectral envelopes; and Cohen 2025 externally validated a commercial multispecialty deep learning platform (Aisap.ai). The three ECG‐based studies exclusively used residual or standard convolutional neural networks trained on raw 12‐lead signals (Lin 2024, Cinq‐Mars 2025, Liang 2025). Six of the eight studies incorporated external or transnational validation cohorts, strengthening generalizability despite architectural and modality heterogeneity. These baseline clinical and technical differences substantially contributed to the high statistical heterogeneity observed in the meta‐analysis.

### 3.3 Pooled Performance of Echocardiography‐Based Models

**Figure 2:**
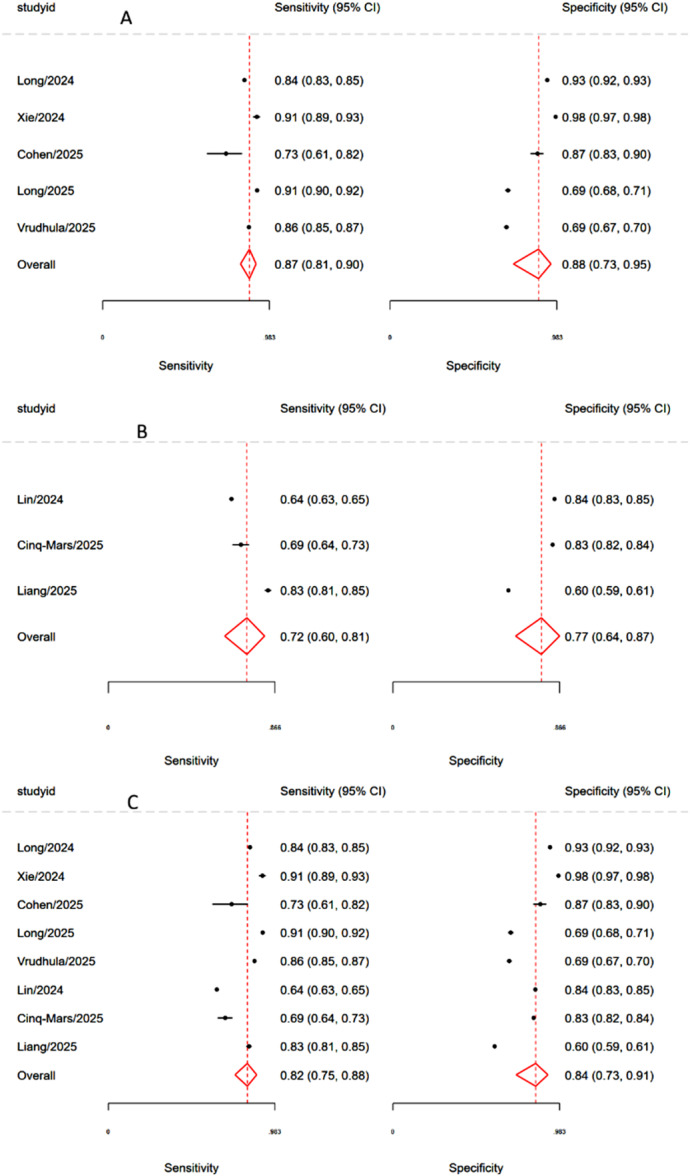
Forest plot showing study‐level and pooled sensitivity and specificity estimates for AI‐based detection of moderate‐or‐greater TR (grade ≥ 2/4 or ≥ moderate by guideline criteria). (A) Echocardiography‐based models. (B) ECG‐based models. (C) Overall assessment combining all model types.

**Figure 3:**
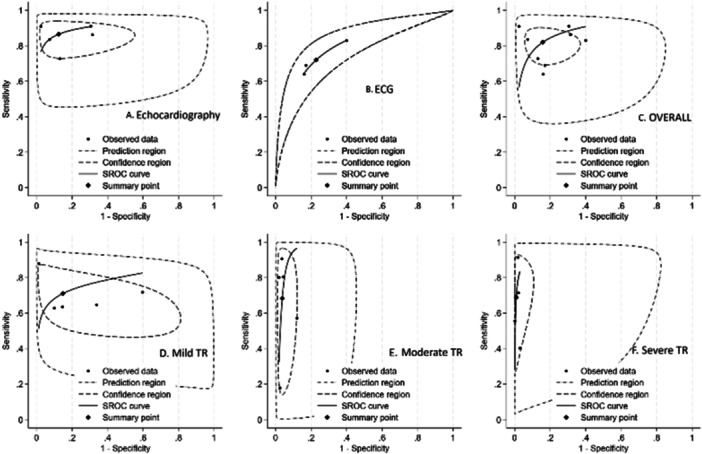
Summary receiver operating characteristic (SROC) plots for AI‐based detection of moderate‐or‐greater TR (grade ≥ 2/4 or ≥ moderate by guideline criteria). (A) SROC curve for echocardiography‐based models. (B) SROC curve for ECG‐based models. (C) Combined SROC plot presenting overall diagnostic performance across all included models. (D) SROC curve for echocardiography‐based models detecting mild TR (grade 1/4 or mild by guideline criteria). (E) SROC curve for echocardiography‐based models detecting moderate TR (grade 2/4 or moderate by guideline criteria). (F) SROC curve for echocardiography‐based models detecting severe TR (grade 3–4/4 or severe/torrential by guideline criteria).

Five studies evaluated the accuracy of echocardiography‐based models for predicting TR. The pooled sensitivity and specificity were 0.87 (95% CI: 0.81–0.90) and 0.88 (95% CI: 0.73–0.95), respectively (Figure 2A), with a summary area under the AUROC of 0.92 (95% CI: 0.89–0.94) (Figure 3A). Between‐study heterogeneity was substantial for the generalized effect (I^2^ = 96.04%), sensitivity (I^2^ = 86.81%), and specificity (I^2^ = 98.90%), reflecting considerable variability in study results.

Subgroup analysis was performed according to TR severity as assessed by echocardiography. For mild TR, pooled sensitivity and specificity were 0.71 (95% CI: 0.61–0.80) and 0.85 (95% CI: 0.58–0.96), respectively, with AUROC of 0.79 (0.76–0.83) (Figure 3D) and high heterogeneity (I^2^: 96.03% overall; 90.91% for sensitivity; 98.97% for specificity) (Supporting Figure 1A). In moderate TR, sensitivity was 0.68 (95% CI: 0.40–0.87) and specificity 0.96 (95% CI: 0.94–0.98), with AUROC of 0.96 (0.94–0.98) (Figure 3E) and I^2^ of 95.42% overall (97.47% for sensitivity; 91.85% for specificity) (Supporting Figure 1B). For severe TR, sensitivity was 0.69 (95% CI: 0.50–0.83) and specificity 0.99 (95% CI: 0.97–1.00), with AUROC of 0.97 (0.95–0.98) (Figure 3F) and lower heterogeneity (I^2^: 90.81% overall; 94.92% for sensitivity; 84.84% for specificity) (Supporting Figure 1C). These findings suggest consistent diagnostic performance across TR severity, with specificity improving as severity increases.

Meta‐regression was performed to investigate study‐level factors contributing to heterogeneity among echocardiography‐based diagnostic models. Sample size (log‐transformed) was not associated with variability in effect size (LRT *χ* ^2^ = 2.03, *p* = 0.36), and the number of validation datasets similarly showed no significant influence (LRT *χ* ^2^ = 3.03, *p* = 0.22), accounting for only modest heterogeneity (I^2^ = 34%, 95% CI: 0%–100%). In contrast, event rate demonstrated a significant association with effect size (LRT *χ* ^2^ = 11.93, *p* < 0.001) and explained a substantial proportion of the between‐study heterogeneity (I^2^ = 83%, 95% CI: 65%–100%) (Supporting Table 3).

### 3.4 Pooled Performance of ECG‐Based Models

A total of three studies evaluated the performance of ECG‐based models for predicting TR. Across these studies, pooled sensitivity and specificity were 0.72 (95% CI: 0.60–0.81) and 0.77 (95% CI: 0.64–0.87), respectively (Figure 2B), with an AUROC of 0.805 (Figure 3B). Between‐study heterogeneity was low for the generalized effect (I^2^ = 3.15%), but substantial for sensitivity (I^2^ = 97.63%) and specificity (I^2^ = 99.72%), suggesting considerable variability in test performance across individual studies.

ECG‐based models did not stratify patients by TR severity; therefore, no subgroup analysis was performed. Additionally, the limited number of studies (*n* = 3) resulted in unstable meta‐regression estimates.

### 3.5 Overall Pooled Performance

The pooled sensitivity and specificity for the overall eight studies were 0.82 (95% CI: 0.75–0.88) and 0.84 (95% CI: 0.73–0.91), respectively (Figure 2C), with a summary area under the AUROC of 0.89 (95% CI: 0.86–0.92) (Figure 3C). Considerable between‐study heterogeneity was observed for the generalized effect (I^2^ = 98.26%), sensitivity (I^2^ = 95.63%), and specificity (I^2^ = 99.33%), suggesting substantial variability in test performance across the included studies. Meta‐regression was conducted to explore potential sources of heterogeneity in the diagnostic performance. Imaging modality was significantly associated with variation in effect size (LRT *χ* ^2^ = 6.23, *p* = 0.04), explaining 68% of the between‐study heterogeneity (I^2^ range: 28%–100%). In contrast, validation type (LRT *χ* ^2^ = 2.60, *p* = 0.27), sample size (log‐transformed; LRT *χ* ^2^ = 2.07, *p* = 0.36), and event rate (LRT *χ* ^2^ = 0.55, *p* = 0.76) were not significantly related to heterogeneity, with low to negligible I^2^ values (Supporting Table 2).

Summary of diagnostic performance across model types is shown in Table 2.

**Table 2:** Pooled diagnostic performance metrics across echocardiography‐based, ECG‐based, and combined models.

Model/Subgroup	No. of studies	Sensitivity (95% CI)	Specificity (95% CI)	AUROC (95% CI)	Heterogeneity (I^2^, overall)
Echocardiography‐based models (overall)	5	0.87 (0.81–0.90)	0.88 (0.73–0.95)	0.92 (0.89–0.94)	96.04%
— Mild TR	5	0.71 (0.61–0.80)	0.85 (0.58–0.96)	0.79 (0.76–0.83)	96.03%
— Moderate TR	5	0.68 (0.40–0.87)	0.96 (0.94–0.98)	0.96 (0.94–0.98)	95.42%
— Severe TR	5	0.69 (0.50–0.83)	0.99 (0.97–1.00)	0.97 (0.95–0.98)	90.81%
ECG‐based models	3	0.72 (0.60–0.81)	0.77 (0.64–0.87)	0.805	3.15%
Overall pooled models	8	0.82 (0.75–0.88)	0.84 (0.73–0.91)	0.89 (0.86–0.92)	98.26%

### 3.6 Sensitivity Analysis: Studies With External Validation

To evaluate whether external validation influenced diagnostic performance and heterogeneity, we conducted a sensitivity analysis restricted to the four studies that reported external validation cohorts (Xie 2024, Cohen 2025, Vrudhula 2025, Liang 2025). Pooled sensitivity was 0.85 (95% CI: 0.80–0.90) and specificity was 0.84 (95% CI: 0.61–0.95), both slightly higher than the primary analysis estimates (0.84 and 0.81, respectively). Notably, heterogeneity in sensitivity decreased substantially (I^2^ = 79.93%) compared to the primary analysis (I^2^ = 94.99%), suggesting that externally validated models exhibited more consistent sensitivity across studies. However, heterogeneity in specificity remained extremely high (I^2^ = 99.09%), and the generalized I^2^ remained elevated at 94.24% (Supporting Figure 2).

### 3.7 Leave‐One‐Out Sensitivity Analysis

A leave‐one‐out sensitivity analysis was conducted to evaluate the stability of the pooled diagnostic performance and effect size. Sequential omission of each study resulted in minimal changes in sensitivity (range: 0.801–0.839) and specificity (range: 0.799–0.864), with heterogeneity remaining high for both sensitivity (I^2^: 98.87%–99.52%) and specificity (I^2^: 99.79%–99.92%). Similarly, the pooled effect size remained robust, ranging from 2.773 to 3.346 with all 95% confidence intervals excluding the null and *p*‐values < 0.001, while the overall pooled effect size was 3.175 (95% CI: 2.258–4.092). These results indicate that no single study disproportionately influenced the meta‐analysis outcomes, confirming the reliability of the findings (Supporting Table 3) (Supporting Figure 3).

### 3.8 Sensitivity Analysis Excluding Conference Abstracts

To assess the robustness of our findings, we performed a sensitivity analysis excluding the two conference abstracts (Long 2024, Cinq‐Mars 2025), leaving six peer‐reviewed full‐text articles. The pooled sensitivity remained stable at 0.83 (95% CI: 0.75–0.89) compared to 0.84 in the primary analysis, while pooled specificity was 0.82 (95% CI: 0.66–0.92) vs. 0.81 in the primary analysis. However, heterogeneity remained extremely high across all metrics (generalized I^2^ = 98.11%, sensitivity I^2^ = 94.99%, and specificity I^2^ = 99.31%), indicating that the exclusion of conference abstracts did not materially reduce between‐study variability (Supporting Figure 4).

### 3.9 Publication Bias

**Figure 4:**
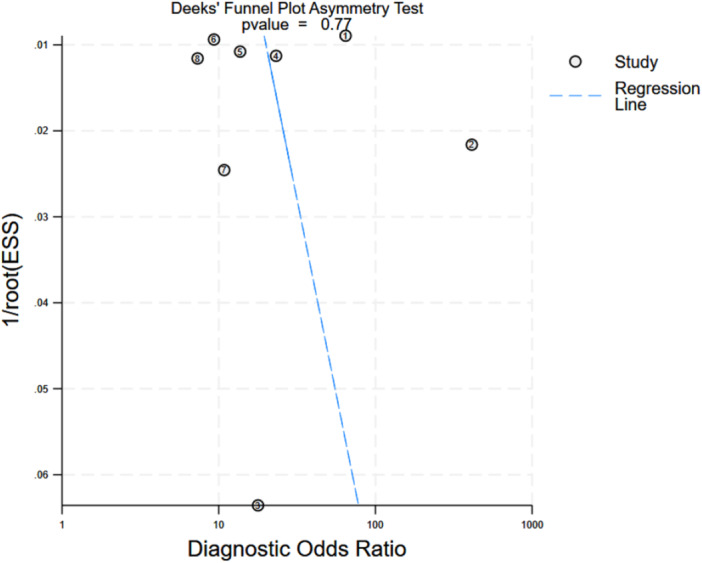
Deeks' funnel plot asymmetry test.

Publication bias was assessed using Deeks' funnel plot asymmetry test, which yielded a *p*‐value of 0.766, indicating no significant evidence of publication bias (Figure 4). However, the small number of included studies and the nature of the machine learning models evaluated may still influence the risk of bias. Therefore, while the test suggests minimal publication bias, it cannot completely rule out the possibility that selective reporting or study characteristics could affect the overall results.

### 3.10 Quality Assessment

Quality appraisal using the PROBAST + AI framework demonstrated that most included studies were methodologically sound, with generally low concerns across development, evaluation, and applicability domains. However, two studies were conference abstracts with insufficient methodological reporting, preventing formal assessment of bias or applicability. These two studies were therefore categorized as no information across all 15 PROBAST + AI domains. Detailed PROBAST + AI domains are in Supporting Table 4.

Across the development domains (participants, predictors, outcomes, analyses, and overall development), six studies were consistently rated as low risk. These studies clearly defined their development cohorts, applied standardized inclusion and exclusion criteria, used appropriate preprocessing methods, and adhered to guideline‐based TR severity definitions. Model development pipelines, including algorithm selection, cross‐validation procedures, and hyperparameter tuning, were generally transparent and avoided data leakage.

Two studies received moderate ratings in one or more development subdomains, typically due to incomplete reporting of uncertainty metrics, limited explanation of model selection rationale, or unclear handling of missing data. In contrast, the two conference‐derived studies lacked basic methodological detail, including unavailable descriptions of population characteristics, predictor measurement, outcome adjudication, or model training processes, resulting in no information ratings.

Evaluation quality representativeness of test datasets, consistency in predictor measurement, standardized outcome application, and appropriateness of validation analyses, was also rated low risk in five to six studies. These studies used evaluation samples that reflected the target TR population and reported key diagnostic metrics (e.g., sensitivity, specificity, and AUROC) with uncertainty intervals.

Moderate risk ratings were assigned to one to two studies due to unclear validation cohort composition or insufficient detail regarding whether test sets were fully independent of training data. As with the development domains, the two conference papers did not provide sufficient information to determine the representativeness of evaluation cohorts or the rigor of validation procedures, leading to no information in all evaluation categories.

Applicability across participants, predictors, outcomes, and analytical methods was judged to be low risk in four to six studies, reflecting strong alignment with real‐world clinical practice. These studies used clinically relevant populations, standard echocardiographic or ECG predictors, and outcomes consistent with guideline‐based TR grading.

**Figure 5:**
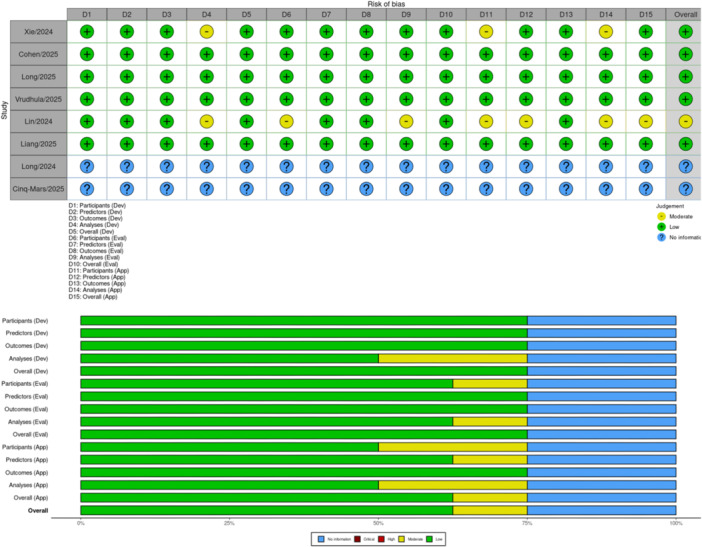
Traffic light plot based on quality assessment.

Moderate risk ratings were assigned in a small subset of studies whose patient populations were narrowly defined or whose predictors required advanced image processing pipelines not widely available in routine practice. Again, the two conference abstracts lacked sufficient detail on clinical populations, predictor accessibility, or outcome definitions, resulting in no information across all applicability domains (Figure 5). Summary of Risk of Bias and Applicability Assessment (PROBAST + AI) is available in Supporting Table 5.

### 3.11 GRADE Assessment

The certainty of evidence across included studies was rated as moderate. Despite the generally low risk of bias and good model performance in both ECG‐ and echocardiography‐based AI models, the overall certainty was tempered by high heterogeneity across studies and variability in study design, population characteristics, and outcome definitions (Supporting Table 6).

## 4 Discussion

The findings from this systematic review and meta‐analysis underscore the emerging role of AI and ML models in enhancing the diagnostic accuracy of TR, a valvular condition historically plagued by underdiagnosis and interobserver variability. Our pooled analysis revealed good diagnostic performance, with echocardiography‐based models achieving a sensitivity of 0.87 (95% CI: 0.81–0.90) and specificity of 0.88 (95% CI: 0.73–0.95), yielding an AUROC of 0.92 (95% CI: 0.89–0.94). ECG‐based models, while slightly less performant (sensitivity 0.72, 95% CI: 0.60–0.81; specificity 0.77, 95% CI: 0.64–0.87; AUROC 0.805), offer a non‐invasive, cost‐effective alternative for initial screening. Overall, the aggregated AUROC of 0.89 (95% CI: 0.86–0.92) positions AI as a promising adjunct to conventional imaging, potentially addressing the diagnostic gaps in TR.

The high heterogeneity (I^2^ > 90%) observed in our meta‐analysis substantially limits the clinical interpretability of pooled estimates. This heterogeneity likely reflects multiple sources: (1) variation in TR severity definitions and grading schemes across studies; (2) differences in disease prevalence and case‐mix, with some studies enriching datasets with higher proportions of moderate‐severe TR; (3) heterogeneity in AI model architectures, training strategies, and input features; and (4) differences between echocardiography‐based models (detecting anatomic TR) and ECG‐based models (inferring TR from electrical patterns). Given this heterogeneity, our pooled estimates should be interpreted as exploratory rather than definitive, and caution is warranted in generalizing these findings to specific clinical contexts.

Although all studies referenced standard echocardiographic TR criteria, differences in how regurgitant jet area, vena contracta width, hepatic vein flow, and Doppler profiles were weighted may have contributed to inconsistent classification of borderline or intermediate cases. Second, variability in imaging protocols, including vendor differences, frame rates, depth settings, and color Doppler gain, introduced additional noise that AI models may handle differently. Studies leveraging raw color Doppler videos, CW Doppler envelopes, or multi‐view apical imaging represent fundamentally different input domains, each with distinct signal‐to‐noise profiles and susceptibility to artifacts. These factors inherently limit direct comparability across AI architectures. Moreover, dataset size imbalance was marked, ranging from a few thousand images in single‐center studies to more than 2 million frames in large multi‐institutional pipelines datasets tend to yield more robust, generalizable models, whereas smaller datasets are more prone to overfitting and inflated internal performance. Variation in model structure (traditional ML vs. 3D CNNs vs. hybrid transformer architectures), the use of internal‐only vs. external validation, and differences in event rates across cohorts further amplified heterogeneity.

Comparative analysis of the included studies reveals two primary AI modalities: echocardiography‐derived approaches (e.g., color Doppler or continuous wave [CW] spectra) and ECG‐based models. Echocardiography‐focused studies, such as those by Vrudhula et al. (2025) and Long et al. (2025a,b; DELINEATE‐Regurgitation), leverage direct imaging of regurgitant jets, achieving superior diagnostic precision. Vrudhula et al.'s automated pipeline, trained on 2 079 898 videos from 47 312 studies, identified apical 4‐chamber views with color Doppler across the tricuspid valve with near‐perfect AUROC (1.000) and detected moderate/severe TR with AUROCs of 0.928 (internal) and 0.951 (external), demonstrating strengths in scalability and external validation across geographically distinct cohorts (Cedars‐Sinai and Stanford) [16]. Similarly, Long et al.'s DELINEATE system, utilizing 1 203 980 color Doppler videos from 93 065 transthoracic echocardiograms (TTEs), reported weighted Cohen's kappa of 0.847 for TR grading and AUROCs exceeding 0.98 for detecting moderate or greater TR, with exact cardiologist agreement in 77.7% of cases [12]. Strengths here include integration of spatiotemporal features via hybrid convolutional neural networks and transformers, enabling nuanced severity classification (6‐grade scale) and risk stratification for progression (e.g., hazard ratios [HRs] up to 7.6 for future moderate‐severe TR in mild cases). Xie et al. (2024) extended this to CW Doppler spectra, segmenting and classifying spectra from 11 654 patients with AUROCs of 0.88 (mild), 0.84 (moderate), and 0.89 (severe) TR internally, validated externally on 573 cases. This approach's strength lies in automating quantitative metrics like jet velocity, reducing operator variability—a key limitation in manual echocardiography [13].

In contrast, ECG‐based models, as in Cinq‐Mars et al. (2025), Liang et al. (2025), and Lin et al. (2024), prioritize accessibility for population‐level screening. Cinq‐Mars et al.'s convolutional neural network, trained on 27 689 paired ECG‐TTE studies, achieved an AUROC of 0.851 for TR detection, with 69.6% sensitivity and 83.4% specificity, highlighting strengths in identifying subtle electrical signatures of right ventricular overload [18]. Liang et al.'s model, developed on 988 618 ECGs from 400 882 patients, predicted future moderate/severe TR with a C‐index of 0.793 and HR of 9.9 for high‐risk quartiles, validated transnationally (China to USA), emphasizing prognostic utility and association with subclinical chamber remodeling [19]. Lin et al.'s multi‐VHD model, using 122 728 ECGs, reported AUROCs > 0.77 for TR, with 37.5%–51.7% of false positives revealing significant echocardiographic findings predictive of progression. These ECG models' strengths include low cost, ubiquity, and ability to forecast disease, potentially guiding targeted echocardiography in resource‐limited settings [17].

However, direct comparisons reveal trade‐offs. Echocardiography‐based models generally outperform ECG counterparts in diagnostic AUROCs (0.92–0.98 vs. 0.80–0.85 for TR), reflecting their proximity to the pathophysiological source (regurgitant flow visualization). Yet, they are constrained by image quality issues, such as acoustic shadowing or poor windows, as noted in Cohen et al. (2025), where only 38% of 1541 TTEs yielded predictions due to mismatched acquisition protocols, underscoring a weakness in real‐world generalizability. External validation in Cohen et al. achieved AUROCs of 0.96 for TR but highlighted annotation bias from clinician‐labeled data [14]. Similarly, high heterogeneity in our meta‐analysis stemmed from varying reference standards (e.g., guideline‐based vs. quantitative metrics) and study designs (retrospective predominance). ECG models, while broader in applicability, suffer from indirect inference, potentially inflating false positives in low‐prevalence settings, as seen in Lin et al.'s correlation with comorbid VHDs [17]. Common weaknesses across studies include retrospective biases, single‐ or few‐center designs, underrepresentation of diverse demographics (e.g., ethnic minorities), and lack of prospective outcome linkage (e.g., mortality or intervention benefits). Strengths are amplified in multi‐center efforts which mitigate overfitting through external testing.

### 4.1 Clinical Implication

Deep learning–based algorithms trained on color Doppler and multi‐view inputs have demonstrated strong discriminatory ability for detecting clinically significant TR, achieving performance comparable across internal and external validations. By reducing interobserver variability inherent in manual grading, AI‐assisted quantification could expedite referral for advanced therapies such as transcatheter edge‐to‐edge repair and improve early risk stratification in heart failure and pulmonary hypertension. Automated extraction of TR jet velocity has further exhibited accuracy similar to expert interpretation, indicating potential for AI‐based triage to decrease unnecessary right heart catheterization in low‐risk cases.

Despite encouraging progress, substantial uncertainties persist across several domains.

Methodological concerns relate to heterogeneity in training datasets and lack of standardized ground truth. Many models are trained on clinician‐labeled images with variable definitions of TR severity, introducing annotation bias and inconsistent calibration across centers. Differences in imaging protocols, echocardiographic views, and vendor‐specific color Doppler settings further impair generalizability.

Technical challenges include performance degradation in borderline or moderate TR, where subtle Doppler signal loss, acoustic shadowing, or respiratory motion can mislead automated classification. Dependence on high‐quality inputs limits reliability in patients with poor acoustic windows, prosthetic valves, or implantable devices. Failures in automated segmentation or jet tracking remain nontrivial, particularly in real‐world, multi‐vendor datasets.

Clinical concerns center on reproducibility and applicability. Most studies remain retrospective and single‐center, with limited demographic diversity, raising questions about model transportability across different populations and care settings. Inconsistent performance in low‐prevalence cohorts may lead to over‐referral, straining specialist resources. Moreover, the absence of prospective validation linking AI‐derived metrics to hard outcomes such as mortality, HF hospitalization, or procedural benefit, limits clinical credibility.

Regulatory and ethical issues further constrain implementation. Transparent reporting standards such as TRIPOD‐AI and CLAIM are not uniformly applied, hindering reproducibility and external scrutiny [20, 21]. Uncertainty regarding accountability, data governance, and cost‐effectiveness adds to the hesitancy surrounding widespread clinical adoption.

### 4.2 Study Limitations

This systematic review and meta‐analysis has several limitations. First, the evidence base was limited by few eligible studies, most being small single‐center retrospective analyses, which constrained statistical power and generalizability. Second, substantial heterogeneity likely resulted from differences in AI model design, imaging modality, data quality, and reference standard. Third, inconsistent reporting formats, including binary detection and multi‐class grading, hindered comparability. Fourth, all studies used retrospective datasets, limiting causal interpretation. Finally, the predominance of studies from high‐resource academic centers may limit relevance to community or low‐resource settings where TR remains frequently under detected.

### 4.3 Future Directions

Future research should focus on large‐scale, multicenter prospective studies that apply standardized AI development and reporting frameworks such as the TRIPOD‐AI and CLAIM checklists to improve transparency and reproducibility [22]. Establishing consensus reference standards for TR detection that integrate multiparametric echocardiographic criteria with emerging biomarkers or cardiac MRI will help reduce variability in ground truth. External validation in diverse populations, including underrepresented ethnic groups and patients with pacemakers or limited acoustic windows, is needed to confirm generalizability. The development of lightweight AI models suitable for deployment on portable ultrasound devices could broaden access to TR screening in primary care and resource‐limited settings. Hybrid strategies that combine AI‐based detection with automated severity stratification within a unified workflow merit investigation to facilitate clinical decision‐making. Finally, randomized controlled trials assessing the effect of AI‐assisted TR detection on clinical outcomes such as time to intervention, heart failure hospitalization, and survival are essential to demonstrate clinical value and cost‐effectiveness before broad implementation.

## 5 Conclusion

Machine learning models demonstrate promising diagnostic accuracy for detecting tricuspid regurgitation across both ECG‐ and echocardiography‐based modalities. However, the certainty of evidence remains moderate, primarily due to substantial heterogeneity across study designs, populations, imaging inputs, and TR grading thresholds. This variability limits the generalizability of the pooled estimates and underscores that current performance metrics should be interpreted with caution. As a result, larger, prospective, and externally validated studies are needed to confirm the robustness, transportability, and real‐world applicability of ML‐based diagnostic tools for TR.

## Author Contributions

Pooya Eini was primary contributor in the design, implementation, and writing of the manuscript. Mohammad Rezayee, Homa Serpoush, and Milan Kassulke independently assessed articles and extracted data. All authors read and approved the final manuscript.

## Funding

The authors have nothing to report.

## Ethics Statement

The authors have nothing to report.

## Consent

The authors have nothing to report.

## Conflicts of Interest

The authors declare no conflicts of interest.

## Policy on Using ChatGPT and Similar AI Tools

In preparing this article, the authors utilized the Grammarly application to enhance linguistic accuracy and clarity. The manuscript underwent meticulous double‐checking to ensure precision, and the authors assume full responsibility for the integrity and originality of the content presented herein.

## Supporting information

Supporting File

## Data Availability

The data that support the findings of this study are available in the Supplementary Material of this article.
