# Supplementary material for: “Diagnostic Performance of Artificial Intelligence in Evaluating Tricuspid Regurgitation: A Systematic Review and Meta‐Analysis”
Source: Clin Cardiol. 2026 Jun 5;49(6):e70366. doi: 10.1002/clc.70366 (PMC13238691; doi:10.1002/clc.70366)
Supplement: Supplementary file 1 — Supporting File [file CLC-49-e70366-s001.docx]

| **Supplementary Table 1. Search syntax for different databases.** | | |
| --- | --- | --- |
| Database | Syntax | Results |
| Pubmed | ("Tricuspid Valve Insufficiency"[Mesh] OR "Tricuspid Valve Insufficiency"[tiab] OR "Insufficiency, Tricuspid Valve"[tiab] OR "Valve Insufficiency, Tricuspid"[tiab] OR "Tricuspid Incompetence"[tiab] OR "Incompetence, Tricuspid"[tiab] OR "Tricuspid Regurgitation"[tiab] OR "Regurgitation, Tricuspid"[tiab] OR "Tricuspid Valve Incompetence"[tiab] OR "Incompetence, Tricuspid Valve"[tiab] OR "Valve Incompetence, Tricuspid"[tiab] OR "Tricuspid Valve Regurgitation"[tiab] OR "Regurgitation, Tricuspid Valve"[tiab] OR "Valve Regurgitation, Tricuspid"[tiab]) AND ("Artificial Intelligence"[MeSH Terms] OR "Artificial Intelligence" OR "Artificial Intelligence Network" OR "Artificial Intelligence Networks" OR "Machine Intelligence" OR "Machine Learning" OR "Deep Learning" OR "Convolutional Neural Network") | 87 |
| WEB OF SCIENCE | TS=("Tricuspid Valve Insufficiency" OR "Insufficiency, Tricuspid Valve" OR "Valve Insufficiency, Tricuspid" OR "Tricuspid Incompetence" OR "Incompetence, Tricuspid" OR "Tricuspid Regurgitation" OR "Regurgitation, Tricuspid" OR "Tricuspid Valve Incompetence" OR "Incompetence, Tricuspid Valve" OR "Valve Incompetence, Tricuspid" OR "Tricuspid Valve Regurgitation" OR "Regurgitation, Tricuspid Valve" OR "Valve Regurgitation, Tricuspid") AND TS=("Artificial Intelligence" OR "Artificial Intelligence Network" OR "Artificial Intelligence Networks" OR "Machine Intelligence" OR "Machine Learning" OR "Deep Learning" OR "Convolutional Neural Network") | 115 |
| Scopus | TITLE-ABS-KEY("Tricuspid Valve Insufficiency" OR "Insufficiency, Tricuspid Valve" OR "Valve Insufficiency, Tricuspid" OR "Tricuspid Incompetence" OR "Incompetence, Tricuspid" OR "Tricuspid Regurgitation" OR "Regurgitation, Tricuspid" OR "Tricuspid Valve Incompetence" OR "Incompetence, Tricuspid Valve" OR "Valve Incompetence, Tricuspid" OR "Tricuspid Valve Regurgitation" OR "Regurgitation, Tricuspid Valve" OR "Valve Regurgitation, Tricuspid") AND TITLE-ABS-KEY("Artificial Intelligence" OR "Artificial Intelligence Network" OR "Artificial Intelligence Networks" OR "Machine Intelligence" OR "Machine Learning" OR "Deep Learning" OR "Convolutional Neural Network") | 219 |
| EMBASE | ('tricuspid valve insufficiency'/exp OR 'tricuspid valve insufficiency':ti,ab OR 'insufficiency, tricuspid valve':ti,ab OR 'valve insufficiency, tricuspid':ti,ab OR 'tricuspid incompetence':ti,ab OR 'incompetence, tricuspid':ti,ab OR 'tricuspid regurgitation':ti,ab OR 'regurgitation, tricuspid':ti,ab OR 'tricuspid valve incompetence':ti,ab OR 'incompetence, tricuspid valve':ti,ab OR 'valve incompetence, tricuspid':ti,ab OR 'tricuspid valve regurgitation':ti,ab OR 'regurgitation, tricuspid valve':ti,ab OR 'valve regurgitation, tricuspid':ti,ab) AND ('Artificial Intelligence':ti,ab,kw OR 'Artificial Intelligence Network':ti,ab,kw OR 'Artificial Intelligence Networks':ti,ab,kw OR 'Machine Intelligence':ti,ab,kw OR 'Machine Learning':ti,ab,kw OR 'Deep Learning':ti,ab,kw OR 'Convolutional Neural Network':ti,ab,kw) | 243 |
| Cochrane Library | TI,AB("Tricuspid Valve Insufficiency" OR "Insufficiency, Tricuspid Valve" OR "Valve Insufficiency, Tricuspid" OR "Tricuspid Incompetence" OR "Incompetence, Tricuspid" OR "Tricuspid Regurgitation" OR "Regurgitation, Tricuspid" OR "Tricuspid Valve Incompetence" OR "Incompetence, Tricuspid Valve" OR "Valve Incompetence, Tricuspid" OR "Tricuspid Valve Regurgitation" OR "Regurgitation, Tricuspid Valve" OR "Valve Regurgitation, Tricuspid") AND TI,AB("Artificial Intelligence" OR "Artificial Intelligence Network" OR "Artificial Intelligence Networks" OR "Machine Intelligence" OR "Machine Learning" OR "Deep Learning" OR "Convolutional Neural Network") | 3 |
| Total |  | 667 |

| **Supplementary table 2. Meta regression analysis.** | | | |
| --- | --- | --- | --- |
| **Parameter** | **LRT χ²** | **P-value** | **I² (%)** |
| **Echocardiography-based models** | | | |
| Log(sample) | 2.03 | 0.36 | 1 |
| Validation type | 3.03 | 0.33 | 34 |
| Event rate | 11.93 | 0.00 | 83 |
| **Overall models** | | | |
| Imaging modality | 6.23 | 0.04 | 68 |
| Validation type | 2.60 | 0.27 | 23 |
| Log(sample) | 2.07 | 0.36 | 3 |
| Event rate | 0.55 | 0.76 | 0 |

| **Supplementary table 3. leave-one-out sensitivity analysis.** | | | | |
| --- | --- | --- | --- | --- |
| Study removed | Sensitivity | Specificity | I2_Sens | I2_Spec |
| Long/2024 | 0.817 | 0.824 | 99.4 | 99.9 |
| Xie/2024 | 0.802 | 0.799 | 99.42 | 99.81 |
| Cohen/2025 | 0.829 | 0.837 | 99.52 | 99.92 |
| Long/2025 | 0.801 | 0.857 | 99.31 | 99.89 |
| Vrudhula/2025 | 0.812 | 0.858 | 99.42 | 99.89 |
| Lin/2024 | 0.839 | 0.842 | 98.87 | 99.91 |
| Cinq-Mars/2025 | 0.834 | 0.843 | 99.44 | 99.9 |
| Liang/2025 | 0.817 | 0.864 | 99.51 | 99.79 |

| 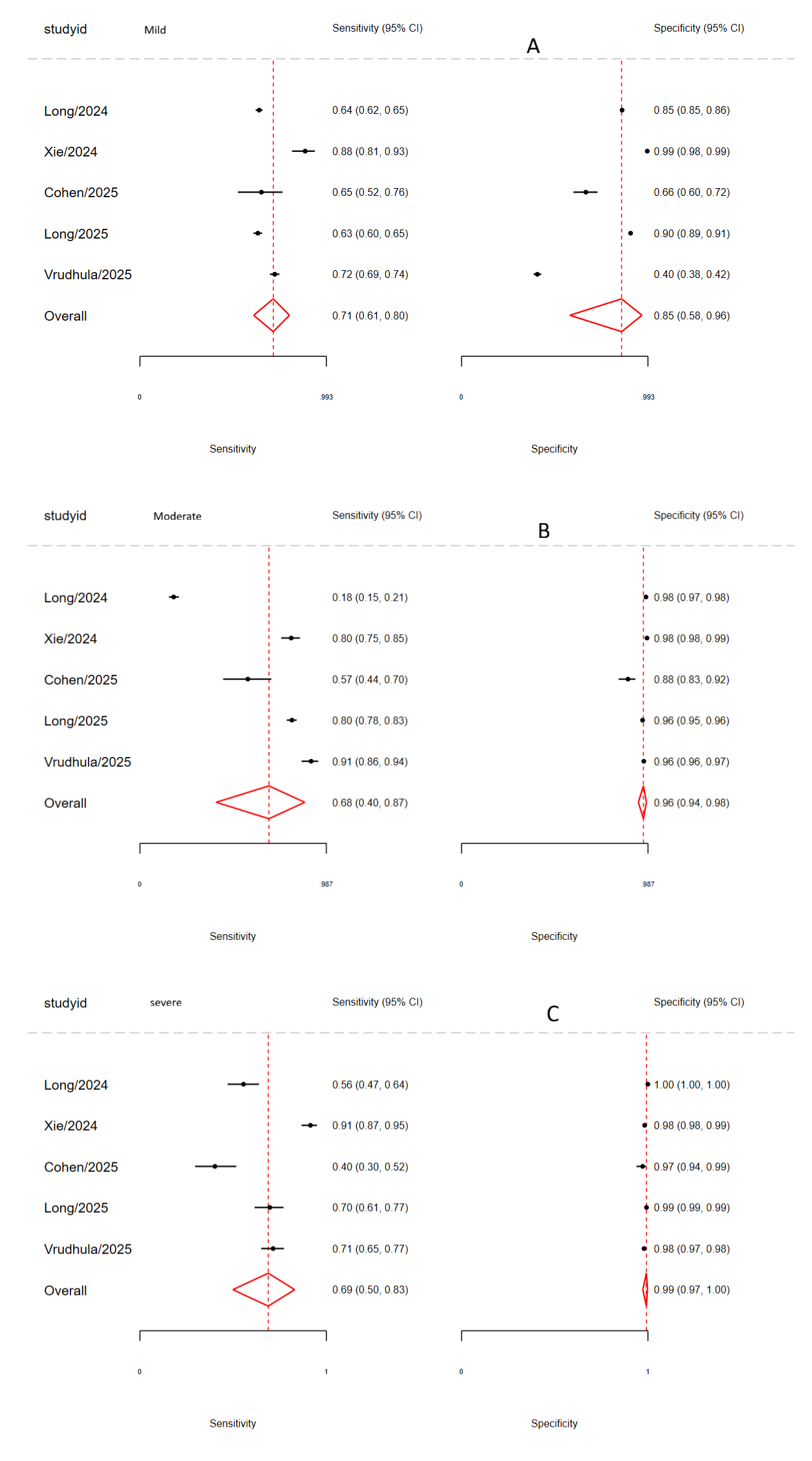 |
| --- |
| **Supplementary figure 1. Forest plot for subgroup analysis based on TR severity. Panel A: Mild TR (grade 1/4 or mild by guideline criteria). Panel B: Moderate TR (grade 2/4 or moderate by guideline criteria). Panel C: Severe TR (grade 3–4/4 or severe/torrential by guideline criteria).** |
|  |
| \| **Supplementary figure 2. Forest plot for Subgroup analysis for studies used external validation.** \| \| --- \| |
| 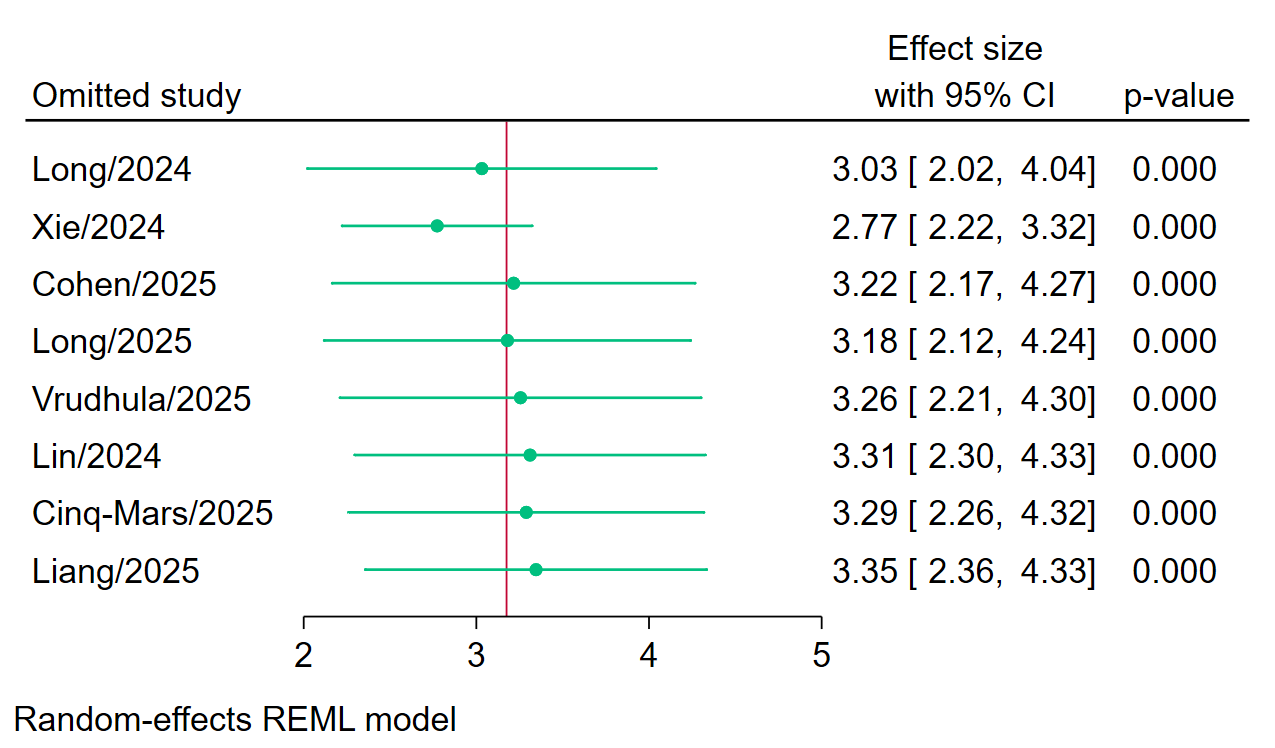 |
| **Supplementary figure 3. leave-one-out sensitivity analysis.** |
| **** |
| **Supplementary figure 4. Sensitivity Analysis Excluding Conference Abstracts.** |
|  |

| **Supplementary table 4. quality assessment check list based on PROBAT+AI tool.** |
| --- |
| **Development (Dev) Domains**  **Participants (Dev)**   1. Were participants in the development dataset representative of the target population (e.g., patients with suspected or diagnosed tricuspid regurgitation), with clearly defined inclusion/exclusion criteria and sufficient sample size for AI model development?   **Predictors (Dev)** 2. Were predictors (e.g., echocardiographic features, clinical variables) clearly defined, consistently measured, and appropriately processed (e.g., normalization, handling of missing data) without knowledge of the outcome to avoid bias in model development?  **Outcomes (Dev)** 3. Was the outcome (tricuspid regurgitation, based on standardized echocardiographic or guideline-defined criteria) clearly defined and assessed independently of predictors to minimize misclassification bias?  **Analyses (Dev)** 4. Were AI model development methods (e.g., algorithm selection, cross-validation, hyperparameter tuning) appropriate, transparent, and free from data leakage or overfitting, with performance metrics reported alongside uncertainty measures?  **Overall (Dev)** 5. Does the development process align with the study question, ensuring the model’s applicability to the target population, predictors, and outcomes for TR assessment?  **Evaluation (Eval) Domains**  **Participants (Eval)** 6. Were participants in the evaluation dataset representative of the target population for TR assessment, with consistent inclusion/exclusion criteria and sufficient diversity to ensure robust performance assessment?  **Predictors (Eval)** 7. Were predictors in the evaluation dataset measured consistently with those in the development dataset, using appropriate preprocessing and without knowledge of the outcome, to ensure unbiased performance estimates?  **Outcomes (Eval)** 8. Was the outcome in the evaluation dataset defined and assessed using the same standardized TR criteria as in development, independently of predictors, to ensure valid performance evaluation?  **Analyses (Eval)** 9. Were evaluation methods (e.g., external validation, performance metrics such as sensitivity, specificity, AUC) appropriate, independent of development data, and reported with uncertainty measures?  **Overall (Eval)** 10. Does the evaluation process align with the study question, ensuring the model’s performance is applicable to the target population, predictors, and outcomes for TR assessment?  **Applicability (App) Domains**  **Participants (App)** 11. Does the study’s participant population (e.g., age, sex, clinical setting) match the intended target population for TR assessment in clinical practice?  **Predictors (App)** 12. Do the study’s predictors (e.g., echocardiographic imaging features, clinical variables) align with those available in routine practice settings, ensuring feasibility of TR assessment in real-world contexts?  **Outcomes (App)** 13. Does the study’s outcome definition align with established clinical standards and echocardiographic guidelines for TR assessment?  **Analyses (App)** 14. Do the study’s analysis methods (e.g., diagnostic accuracy metrics, subgroup analyses) support clinical decision-making needs for TR assessment in diverse settings?  **Overall (App)** 15. Does the study’s design and findings align with the study question, ensuring applicability to the target population, predictors, outcomes, and clinical contexts for TR assessment? |

| **Supplementary Table 5: Summary of Risk of Bias and Applicability Assessment (PROBAST+AI)** | | | | | |
| --- | --- | --- | --- | --- | --- |
| **Study** | **Domain 1: Participants** | **Domain 2: Predictors** | **Domain 3: Outcome** | **Domain 4: Analysis** | **Overall Risk** |
| **Long/2024** | Low risk: Large cohort of 93,065 TTEs with clear train/validation/test split | Low risk: Standardized color Doppler video clips from TTE, measured independently of outcome | Low risk: TR severity graded per standard criteria (mild 24%, moderate 7%, severe 1%) | Low risk: Hybrid neural network (CNN + transformer) with appropriate data partitioning | Low risk |
| **Xie/2024** | Low risk: Large development cohort (11,654 patients) with both internal (1,500) and external (573) validation | Low risk: Standardized CW Doppler spectra images, measured independently of outcome | Low risk: TR severity clearly defined (mild 5%, moderate 10%, severe 8%) | Moderate risk: Deep learning model with validation, but limited reporting of hyperparameter tuning and overfitting mitigation | Low risk |
| **Cohen/2025** | Low risk: Multi-center external validation cohort (1,541 TTEs) from Israel and USA with diverse comorbidities | Low risk: Standardized echocardiographic images, measured independently of outcome | Low risk: TR severity clearly defined (mild 23%, moderate 22%, severe 28%) | Low risk: Deep learning with external validation and comprehensive performance assessment | Low risk |
| **Long/2025** | Low risk: Large single-center cohort (71,660 TTEs) with clear demographics (mean age 61.4, 48.5% male) | Low risk: Standardized color Doppler videos from TTE, measured independently of outcome | Low risk: TR severity clearly defined (mild 23%, moderate 12%, severe 2%) | Low risk: DELINEATE-Regurgitation multiview CNN/transformer with appropriate methodology | Low risk |
| **Vrudhula/2025** | Low risk: Large multi-cohort study (55,323 studies) with development, tuning, internal and external validation; detailed demographics and comorbidities reported | Low risk: Standardized apical 4-chamber color Doppler videos, measured independently of outcome | Low risk: TR severity clearly defined (mild 36%, moderate 6%, severe 6%) | Low risk: Deep learning computer vision with view classifier and severity classifier, comprehensive validation strategy | Low risk |
| **Lin/2024** | Moderate risk: Large cohort (77,047 patients) with internal and external validation, but single-country (Taiwan) limiting generalizability | Low risk: Standardized 12-lead ECG signals, measured independently of outcome | Low risk: TR outcome clearly defined (60% event rate) | Moderate risk: Deep learning model with validation, but limited detail on hyperparameter selection and overfitting prevention | Moderate risk |
| **Long/2024** | No information: Conference abstract with insufficient detail on participant selection, sample size, or criteria | No information: Predictors not clearly described, unclear protocols or preprocessing | No information: Outcome definition and reference standard not specified | No information: AI methodology mentioned but insufficient detail on model, training, or validation | No information |
| **Cinq-Mars/2025** | No information: Conference abstract with minimal detail on cohort characteristics or selection | No information: ECG-based predictors mentioned but insufficient detail on acquisition or processing | No information: TR outcome mentioned but reference standard not clearly defined | No information: Machine learning mentioned but no detail on algorithm, training, or validation | No information |

| **Supplementary table 6. Summary of GRADE Assessment.** | | |
| --- | --- | --- |
| **Domain** | **Assessment** | **Impact on Certainty** |
| Risk of bias | Low | No downgrade |
| Inconsistency (Heterogeneity) | High (I² > 90% in several analyses) | Downgrade by 1 level |
| Indirectness | Low | No downgrade |
| Imprecision | Moderate (limited sample sizes in some studies) | No or minor downgrade |
| Publication bias | Not detected / unclear | No downgrade |
| Overall model performance | Good (pooled sensitivity & specificity high) | Supports moderate certainty |
| **Overall certainty of evidence** | Moderate | — |
